# 2671. The Sexual Health Clinic Mpox Response in King County, Washington, May–October 2022

**DOI:** 10.1093/ofid/ofad500.2282

**Published:** 2023-11-27

**Authors:** Ellora Karmarkar, Chase Cannon, Meena Ramchandani, Matthew R Golden, Julia C Dombrowski

**Affiliations:** University of Washington, Seattle, WA; University of Washington, Seattle, WA; University of Washington, Seattle, WA; University of Washington, Seattle, WA; University of Washington, Seattle, WA

## Abstract

**Background:**

Sexual health clinics, vital to the 2022 mpox outbreak response, were challenged by high patient volumes and delayed access to vaccines and tecovirimat. We describe the Public Health - Seattle King County Sexual Health Clinic (SHC) response, including vaccine and tecovirimat distribution, from 5/23/22 (first case in King County) to 10/14/22.

**Methods:**

SHC began vaccinations in clinic on 7/4/22 and implemented a mass vaccination clinic on 7/25/22. Tecovirimat prescriptions initially required positive test results; by 7/29/22 SHC offered same-day presumptive treatment based on clinical suspicion. We calculated vaccines administered per week, proportion of visits related to mpox evaluation, and collected and summarized demographic and clinical data for diagnosed mpox cases. We assessed temporal trends in treatment type (presumptive vs results-based) using Kruskal-Wallis testing and differences in treatment receipt by race/ethnicity using chi-square testing (p< 0.05).

**Results:**

From 5/23/22 to 10/14/22, SHC provided ≥1 vaccine dose to 9,753 people; the highest number within a week (n=1,537) were provided the week of 8/1/22 (Figure 1). Of 4,845 clinic visits, 486 (10%) patients were evaluated and tested for mpox; 174 (36%) were positive, comprising 36% of cases in King County at the time. 94% were cisgender men; 57% were White. 74 (43%) had severe symptoms including proctitis, hematochezia, or need for adjunctive pain control. Of 172 patients with complete clinical data, 154 (90%) received tecovirimat, including 83 (54%) treated presumptively. Provision of presumptive treatment increased significantly over time (P< 0.001) (Figure 2). Treatment receipt did not differ significantly by race/ethnicity (P=0.4).

**Figure:**
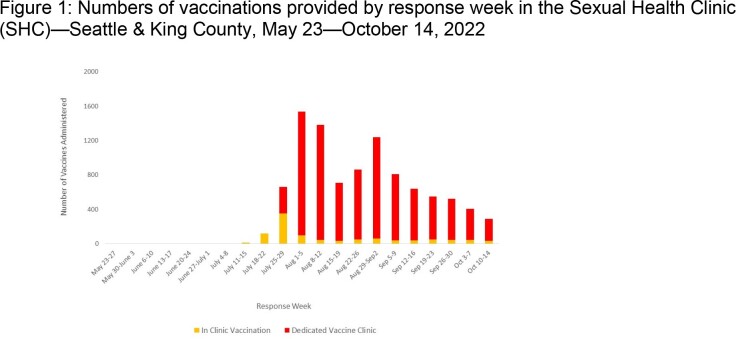

**Figure:**
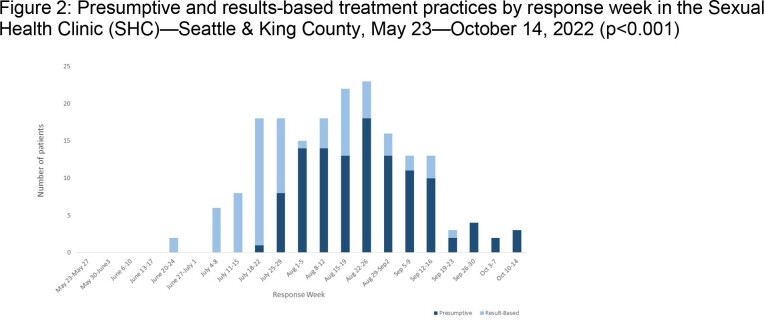

**Conclusion:**

The SHC substantially expanded vaccine access, served over 1/3 of patients with mpox in King County, and expedited mpox treatment through presumptive therapy, while continuing general sexual health services for patients within the county.

**Disclosures:**

**Chase Cannon, MD MPH**, Roche Diagnostics: Advisor/Consultant **Julia C. Dombrowski, MD, MPH**, Hologic: Grant/Research Support|Mayne Pharmaceuticals: Grant/Research Support

